# Socio-environmental drivers and suicide in Australia: Bayesian spatial analysis

**DOI:** 10.1186/1471-2458-14-681

**Published:** 2014-07-04

**Authors:** Xin Qi, Wenbiao Hu, Kerrie Mengersen, Shilu Tong

**Affiliations:** 1 School of Public Health, Xi’an Jiaotong University Health Science Center, Xi’an, Shaanxi 710061, China; 2 School of Public Health and Social Work, Queensland University of Technology, Kelvin Grove, QLD 4059, Australia; 3 Faculty of Science and Engineering, Queensland University of Technology, Brisbane, QLD 4000, Australia

**Keywords:** Suicide, Spatial, Socio-environmental, Australia

## Abstract

**Background:**

The impact of socio-environmental factors on suicide has been examined in many studies. Few of them, however, have explored these associations from a spatial perspective, especially in assessing the association between meteorological factors and suicide. This study examined the association of meteorological and socio-demographic factors with suicide across small areas over different time periods.

**Methods:**

Suicide, population and socio-demographic data (e.g., population of Aboriginal and Torres Strait Islanders (ATSI), and unemployment rate (UNE) at the Local Government Area (LGA) level were obtained from the Australian Bureau of Statistics for the period of 1986 to 2005. Information on meteorological factors (rainfall, temperature and humidity) was supplied by Australian Bureau of Meteorology. A Bayesian Conditional Autoregressive (CAR) Model was applied to explore the association of socio-demographic and meteorological factors with suicide across LGAs.

**Results:**

In Model I (socio-demographic factors), proportion of ATSI and UNE were positively associated with suicide from 1996 to 2000 (Relative Risk (RR)_ATSI_ = 1.0107, 95% Credible Interval (CI): 1.0062-1.0151; RR_UNE_ = 1.0187, 95% CI: 1.0060-1.0315), and from 2001 to 2005 (RR_ATSI_ = 1.0126, 95% CI: 1.0076-1.0176; RR_UNE_ = 1.0198, 95% CI: 1.0041-1.0354). Socio-Economic Index for Area (SEIFA) and IND, however, had negative associations with suicide between 1986 and 1990 (RR_SEIFA_ = 0.9983, 95% CI: 0.9971-0.9995; RR_ATSI_ = 0.9914, 95% CI: 0.9848-0.9980). Model II (meteorological factors): a 1°C higher yearly mean temperature across LGAs increased the suicide rate by an average by 2.27% (95% CI: 0.73%, 3.82%) in 1996–2000, and 3.24% (95% CI: 1.26%, 5.21%) in 2001–2005. The associations between socio-demographic factors and suicide in Model III (socio-demographic and meteorological factors) were similar to those in Model I; but, there is no substantive association between climate and suicide in Model III.

**Conclusions:**

Proportion of Aboriginal and Torres Strait Islanders, unemployment and temperature appeared to be statistically associated with of suicide incidence across LGAs among all selected variables, especially in recent years. The results indicated that socio-demographic factors played more important roles than meteorological factors in the spatial pattern of suicide incidence.

## Background

The impacts of socio-environmental factors on suicide have drawn increasing public attention, especially due to severe long-lasting global climate changes [1]. As climate factors vary across diverse areas, their association with suicide may also be different. A lot of studies have examined how meteorological factors, e.g., temperature [2-9], rainfall [2,7] and sunshine [8] are associated with suicide incidence in different countries. Most studies on meteorological factors and suicide, however, regard each study area as a single homogeneous region and usually apply the meteorological data by using the mean value of meteorological stations within the whole study area, e.g., 64 stations in South Korea [3] or 6 stations in Taiwan [7]. As meteorological factors vary across different stations in the area, even within the same timeframe, and, e.g., temperature between coastal and inland areas, and also vary across time, this approach may ignore the spatial and temporal differences of climate and may adversely influence inferences made about the association between climate and suicide.

From the opposite perspective, Geographical Information System (GIS) and spatial analyses have been widely used in suicide research, e.g. exploring the pattern of suicide across small areas [10-12], identifying high risk geographical clusters [13-17], and examining the associations of socioeconomic and demographic factors with suicide [18-21]. These studies, however, have not explored the association between climate and suicide across different areas. This may be particularly important in large regions with substantial variation in climate zones.

Australia lies in the southern hemisphere and is subject to very wide variation in climatic conditions. Moreover, there is also a wide range of socio-demographic factors such as Aboriginal and Torres Strait Islanders, and unemployment. This provides motivation for exploring potential spatial associations between these factors and suicide. Some Australian studies have explored the association between socio-environmental factors and suicide across different areas [15,22]. For example, Qi et al. examined the association of meteorological -and socio-demographic factors and suicide over Local Governmental Areas in Queensland [15]. This study only covered a relative short period (i.e., from 1999 to 2003), however. Thus it is necessary to examine the associations between socio-environmental factors and suicide over a longer period of time at a small area level. Hanigan et al. explored and identified the impact of drought on suicide by examining databases in different Statistical Divisions (SDs) in New South Wales, Australia over 30 years [22]. They used a relative large geographic scale, however, and did not take into account the spatial autocorrelation of suicide incidence in the neighbouring areas, which may result in bias in assessing the socio-environmental impact on suicide [23]. Similar limitations were also found in one study in Taiwan [24], which examined the socio-environmental drivers on suicide at the county level and found that unemployment outweighed meteorological variables on association with suicide. Thus it is important to examine the association of meteorological and socio-demographic factors with suicide at a small area level over different time periods.

A difficulty with small area analysis of a relatively rare outcome is that the small numbers of cases within areas can lead to unstable and potentially biased estimates of local rates and hence misleading inferences. This can be rectified using spatial smoothing methods that ‘borrow strength’ among neighbouring areas to provide improved estimates. This is based on the assumption that neighbouring areas share common characteristics. This spatial autocorrelation is quite localized, however, and its magnitude can vary across a region. A popular method that utilizes a local smoothing approach is a conditional autoregressive (CAR) model. Inclusion of spatial autocorrelation in the models of meteorological and socio-demographic factors and suicide can help to improve estimates of the associations and quantify the amount of residual spatial correlation that could be explained by other geographically varying factors.

This study aims at using an exploratory spatial analysis on assessing the associations between socio-environmental factors (socio-demographic and meteorological factors) and suicide across local areas over different time period in Australia, with three research questions proposed. First: whether or not socio-environmental factors have significant associations with suicide incidence over different areas. Second: whether or not the association between socio-environmental factors and suicide vary over different time period. Third: whether or not socio-demographic factors have more significant associations with suicide incidence than meteorological factors. Although this is a study of association, not causation, the results of this study can help to improve our understanding of potential indicators of suicide.

## Methods

### Data sources

Suicide data between 1986 and 2005 were obtained from the Australian Bureau of Statistics (ABS), including sex, age, country of birth, date of suicide and Statistical Local Area (SLA) code in eight states and territories: New South Wales (NSW), Victoria (VIC), Queensland (QLD), South Australia (SA), Western Australia (WA), Tasmania (TAS), Northern Territory (NT) and Australian Capital Territory (ACT). Suicide cases were diagnosed using the International Classification of Disease (ICD) Code (ICD 9 before 1999: 950.0-959.9; ICD 10 for 1999 and later: X60-X84). The definitions of suicide before and after 1999 are same and there is no significant implication for the changing of suicide diagnosis. The application for setting access to more detailed information (e.g., SLA code) of suicide data after 2005 is still under review by ABS. Thus, the trend of associations between socio-environmental factors and suicide after 2005 could not be examined from spatial perspective. As the incidence of suicide is generally low and SLA boundaries changed over time, we used Local Governmental Area (LGA) as a spatial scale in this study. Each LGA contains one or more SLAs and LGA boundaries are more stable compared with SLA boundaries. The Australian Standard Geographical Classification (ASGC), published by ABS and updated annually, was used in transferring SLA codes into LGA codes, after adjusting for SLA/LGA boundary changes over time.

The population data, including age, sex, Socio-Economic Indexes for Areas (SEIFA), proportion of Aboriginal and Torres Strait Islanders (ATSI) and unemployment rates at the LGA level were obtained from Census Data (CDATA) for 1986, 1991, 1996, 2001 and 2006 Census by ABS after adjusting for LGA boundary changes over years. SEIFA indicates the general level of socioeconomic status across Australian LGAs, with higher SEIFA values indicating higher socioeconomic development. Population and socio-demographic data in the years outside of census years were interpolated linearly using census data.

Meteorological variables (1985–2005, monthly), including rainfall (mm), relative humidity (%), mean temperature (°C), were provided by the Australian Bureau of Meteorology. The mean values of each climate variable from monitoring stations in each LGA were used in the analysis. Some LGAs (e.g., some urban LGAs with small geographical area) had no station data. To solve this problem, the data from the nearest stations to the centroid (longitude/latitude) of these LGAs were used to represent the meteorological data for these LGAs.

### Data analysis

Suicide rate is the dependent variable while meteorological and socio-demographic (proportion of ATSI, SEIFA and unemployment rate) factors are independent variables in this study. Suicide rate at the LGA level was calculated after adjusting for age groups and sexes. As some rural areas had very few suicide cases, we aggregated the suicide data altogether for table display and into four of 5-year intervals (1986–1990, 1991–1995, 1996–2000 and 2001–2005) for map display. Meteorological (rainfall for yearly mean, temperature and humidity for monthly mean) and socio-demographic (yearly mean) datasets were displayed with suicide rates in the table as above. Correlations of socio-environmental variables and suicide rates across LGAs were also plotted (20 years altogether). A Spearman correlation analysis was applied to check the multicollinearity of the explanatory variables. Those variables with high correlation (r_s_ ≥ |0.80|) were included in separate models.

Bayesian spatial and temporal models have been increasingly used in public health research [11,25-27]. Bayesian smoothing has been used to stabilize estimates in spatiotemporal patterns of diseases in small areas with extremely low population [28]. The Bayesian conditional autoregressive (CAR) model has been used to describe geographical variation in a specific disease risk between spatially aggregated units, such as the administrative divisions of a country [29]. In this study, the Bayesian CAR model with Poisson distribution was used to compare suicide rates and their socio-environmental determinants across LGAs.

The Bayesian CAR model is formulated as follows [30]:

$$\begin{array}{ll} \;\log\left({µ}_{i}\right)\;= & \;\log\left(n_{i}\right)\;+\;\left(\beta_{0}+\beta_{1}X_{1i}+\;\ldots \;+\beta_{m}X_{\mathrm{mi}}\right)\\ & +U_{i}+S_{i} \end{array}$$

In the above formula, *U*_*i*_, *S*_*i*_ follows a Poisson distribution with mean μ_*i*_, *i* refers to the location and *n* to the population. μ_*i*_ represents the mean of the dependent variable, *X* indicates the fixed effect*, β* represents the meteorological variables, SEIFA, and socio-demographic variables. *β*_0_ + *β*_1_*X*_1i_ + … + *β*_m_*X*_mi_ is the regression equation. *U*_*i*_ denotes the unstructured random effects for each of the *i* areas; and *S*_*i*_ represents the structured random effect which is spatially correlated. Four sets of 5-year aggregated datasets (including suicide, population and socio-environmental data) were analysed separately by Besag, York and Mollie (BYM) models with structured and unstructured residuals, to examine if there are differences of socio-environmental variables significantly associated with suicide over various time periods.

Some exploratory data analyses were implemented in the Bayesian CAR model at first. The deviance information criterion (DIC) was checked. A lower DIC indicates the better goodness of fit in the model. Initially, no spatial elements were included. After that unstructured covariance (*U*_*i*_) was added in the model; then structured covariance (*S*_*i*_) were also added. The residuals of the final model were mapped. In the selection of independent variables, we added only socio-demographic variables as Model I and only meteorological variables as Model II. Finally both socio-demographic and meteorological factors were added as Model III. For each of Models I, II and III, both exploratory and final data analyses were included. As some of the SEIFA and demographic variables may have high multicollinary (e.g., r_s_ ≥ |0.80|), each of the high-correlated variables were input in the Bayesian CAR model consequently but not in the same model to select suitable models. Markov Chain Mount Carlo (MCMC) simulation was applied in parameter estimation using a single chain algorithm. MCMC was also used in diagnostics. Each three models were performed with 10,000 iterates for burn-in and 100,000 iterates for running. Convergence was checked by autocorrelations of selected parameters. The WinBUGS package was used to run the Bayesian CAR model. The WinBUGS code in Model III was provided as supplementary file (Additional file 1: File S1). The ethical approval for this project was granted by the Human Research Ethics Committee, Queensland University of Technology.

## Results

**Figure 1:**
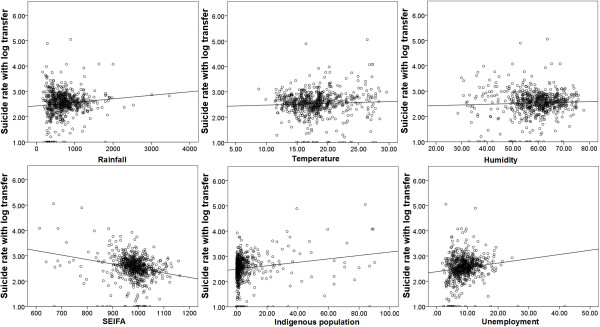
Scatterplot of suicide rate and socio-environmental variables (annual average).

**Figure 2:**
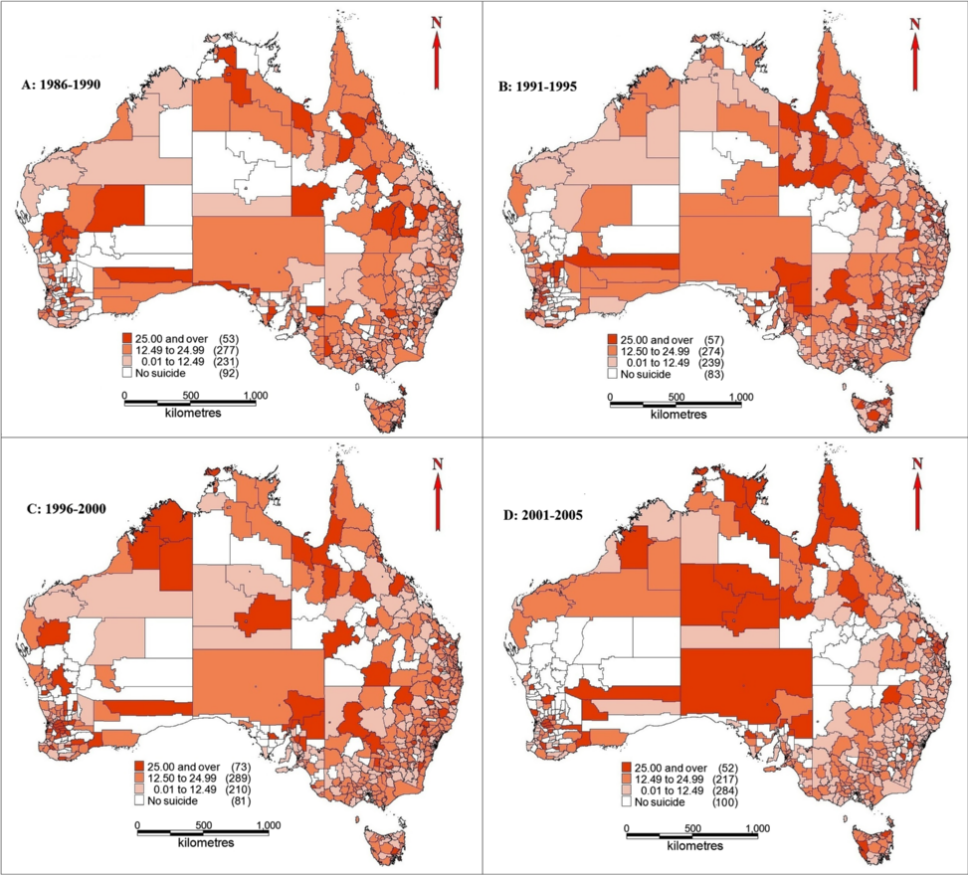
**Suicide rates across LGAs and unincorporated SLAs (1986–2005). A**: 1986-1990; **B**: 1991-1995; **C**: 1996-2000; **D**: 2001-2005.

A total of 45,293 suicide deaths (1986–2005) were included in the analysis. Table 1 shows the statistical distribution of suicide rate and socio-environmental variables using yearly average data at the LGA level. 75% of LGAs had less than 4 suicides occurred annually on average. Some LGAs had a suicide rate over 11-fold above the national suicide rate. Figure 1 demonstrates the crude relationships between socio-environmental factors and suicide over the 20 years period. The plots indicate that rainfall, temperature, proportion of ATSI and unemployment rate were positively associated with suicide rates. SEIFA and humidity had negative associations, however.Figure 2 indicates the distribution of suicide rates at the LGA level. Suicide rates in urban and coastal areas were steady over the whole study period; some rural and remote areas, especially SLAs in NT and SA, however, had higher suicide rates between 1996 and 2005 compared with the previous 10 years. Some LGAs in the southwest of QLD and eastern part of WA had no suicide reported during the whole study period.

**Table 1:** Summary statistics of suicide and socio-environmental variables at the LGA level

	**Mean**	**SD**	**Min**	**Percentiles**			**Max**
				25	50	75	
Suicide rate (per 100,000)	14.00	9.350	0.00	10.37	12.97	16.41	168.28
SEIFA	981.94	64.664	613.31	959.41	987.03	1015.63	1162.11
ATSI (%)	5.91	13.222	0.00	0.76	1.72	4.19	90.22
Unemployment rate (%)	8.89	3.756	1.84	6.31	8.43	10.73	41.77
Rainfall (mm)	720.34	376.665	162.43	448.97	655.98	876.92	3464.77
Temperature (°C)	18.10	3.756	8.75	15.65	17.65	19.98	29.61
Humidity (%)	58.74	8.898	28.54	54.13	60.06	64.45	77.81

Note: SEIFA: Socio-Economic Indexes for Areas in each LGA, score 1000 for the national level. ATSI: Proportion of Aboriginal and Torres Strait Islanders among the total population in each LGA. Unemployment rate: Proportion of unemployed population among the total population in each LGA. Suicide rate, SEIFA, ATSI, unemployment rate and rainfall were presented as yearly average values. Temperature and humidity were presented as monthly average values.

**Figure 3:**
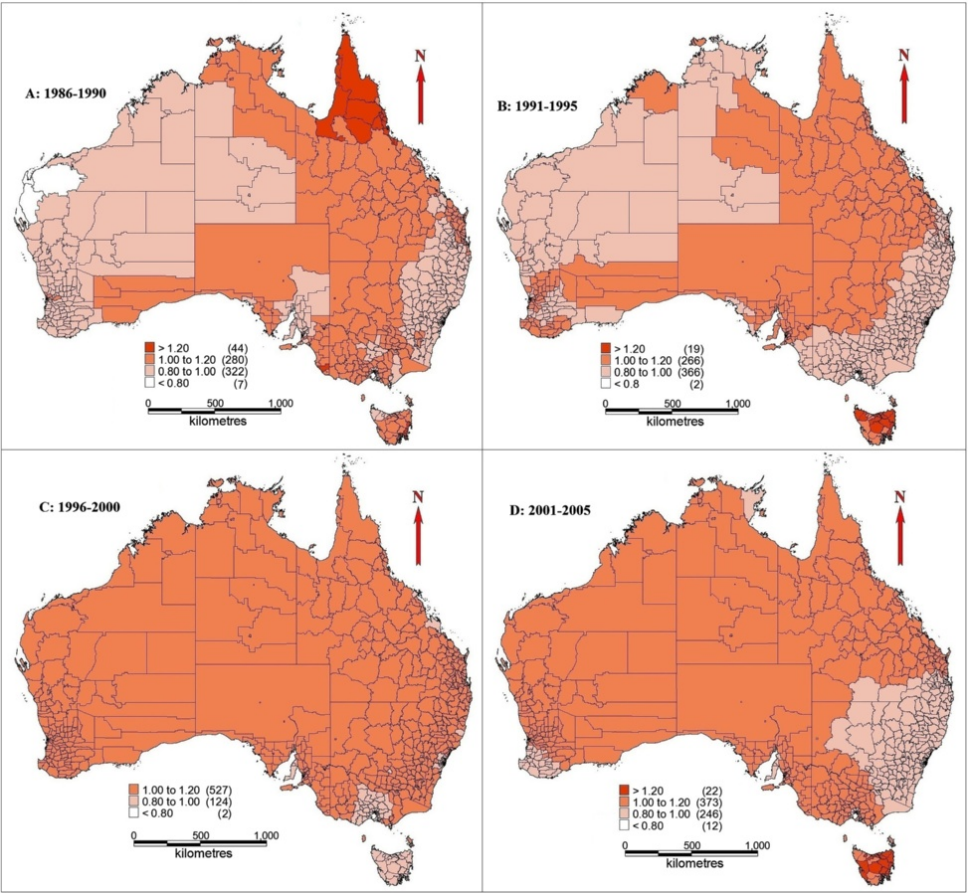
**Spatial random effect for suicide rates (1986–2005, structured spatial residuals with fixed effect in Model III). A**: 1986-1990; **B**: 1991-1995; **C**: 1996-2000; **D**: 2001-2005.

Table 2 provides a summary of the association between socio-environmental factors and suicide based on the Models I, II and III. Model I examines the association between socio-demographic factors and suicide. The tables show the posterior estimates of the relative risk (RR) and corresponding 95% credible intervals (CI). Both SEIFA (RR = 0.9983, 95% CI: 0.9971-0.9995) and percentage of ATSI (RR = 0.9914, 95% CI: 0.9848-0.9980) were slightly and negatively associated with suicide between 1986 and 1990. There was an increase of 1% in suicide incidence for a 1% higher of ATSI proportion between 1996 and 2000 (95% CI: 0.62%, 1.51%), and 2001 to 2005 (95% CI: 0.55%, 1.48%), however. Unemployment was also positively associated with suicide during 1996–2000 (RR = 1.0187, 95 CI: 1.0060-1.0315) and 2001–2005 (RR = 1.0198, 95 CI: 1.0041-1.0354. No significant association of socio-environmental factors with suicide was found between 1991 and 1995. The association between meteorological factors and suicide was examined using Model II. 1°C of increased mean temperature was accompanied with higher suicide rates of 2.27% between 1996 and 2000 (95% CI: 0.73%, 3.82%) and of 3.24% from 2001 to 2005 (95% CI: 1.26%, 5.21%). Climate factors were not significantly associated with suicide between 1986 and 1995. Model III examined the association of both socio-demographic and meteorological factors with suicide. The associations between socio-demographic factors and suicide in Model III were similar to those obtained in Model I from 1986 to 1990 (ATSI proportion: RR = 0.9912, 95% CI: 0.9843-0.9980; SEIFA: RR = 0.9985, 95% CI: 0.9972-0.9997), from 1996 to 2000 (ATSI proportion: RR = 1.0125, 95% CI: 1.0073-1.0177; unemployment: RR = 1.0199, 95% CI: 1.0043-1.0356) and from 2001–2005 (ATSI proportion: RR = 1.0101, 95% CI: 1.0055-1.0148; unemployment: RR = 1.0180, 95% CI: 1.0047-1.0314). No substantive associations between meteorological factors and suicide, however, were discovered in Model III in any of the study periods. Model III had higher DIC value than other two models in all four study periods. DIC value in the study period from 1996 to 2000 was higher than that in other study periods in each model. The final model had lower DIC than exploratory models in each of 5-year period studies and in each of Models I, II, III.Figure 3 indicates the spatial residual variation after taking into account the socio-environmental factors (fixed effect in Model III). The high-risk areas (RR ≥ 1.20) were in north QLD between 1986 and 1990. The dominant cluster in the 1991–1995 time period was in TAS (1991–1995). The majority of WA and NT, southeast QLD and east of NSW had relatively lower risks in the 1986–1995 period. The majority of Australian territory however, was identified as upper middle high risk (1.00 to 1.20) areas from 1996 to 2005, with some low risk areas in south VIC and TAS (1996–2000), and south QLD, southwest of WA, majority of NSW (2001–2005). A high-risk cluster in TAS (2001–2005) was also discovered. Many rural areas (e.g., NT and WA) have a sparse population density, thus even a small number (e.g., 1 or 2) of suicide cases increase in particular rural LGA over time (e.g., from 1991–1995 period to 1996–2000 period) indicates a much higher relative risk increase over time compared with urban areas. The risks in capital cities (e.g., Sydney and Melbourne) were consistent over time and relatively lower than the national level. Other types of models, e.g., those without adding random effect index, were also checked; however, they have higher DIC value. Thus we chose the model as above with results presented.

**Table 2:** Socio-environmental factors and suicide

**Models**	**Time period (DIC)**	**Variables**	**RR (95% CI)**	**MC error**
Model I: Socio-economic factors and suicide	1986-1990 (3148.99)	**SEIFA**	**0.9983 (0.9971, 0.9995)**	**0.00002420**
		**ATSI (%)**	**0.9914 (0.9848, 0.9980)**	**0.00007110**
		Unemployment (%)	0.9980 (0.9818, 1.0141)	0.00035200
	1991-1995 (3174.31)	SEIFA	0.9992 (0.9982, 1.0003)	0.00001820
		ATSI (%)	0.9982 (0.9927, 1.0038)	0.00005980
		Unemployment (%)	1.0075 (0.9929, 1.0221)	0.00028800
	1996-2000 (3276.25)	SEIFA	0.9996 (0.9988, 1.0004)	0.00001170
		**ATSI (%)**	**1.0107 (1.0062, 1.0151)**	**0.00005240**
		**Unemployment (%)**	**1.0187 (1.0060, 1.0315)**	**0.00021000**
	2001-2005 (3140.36)	SEIFA	0.9993 (0.9985, 1.0002)	0.00001070
		**ATSI (%)**	**1.0126 (1.0076, 1.0176)**	**0.00005490**
		**Unemployment (%)**	**1.0198 (1.0041, 1.0354)**	**0.00023800**
Model II: Meteorologic factors and suicide	1986-1990 (3147.70)	Rainfall (100 mm)	0.9966 (0.9814, 1.0119)	0.00030600
		Temperature (°C)	0.9987 (0.9772, 1.0202)	0.00055900
	1991-1995 (3171.05)	Humidity (%)	0.9971 (0.9893, 1.0050)	0.00020900
		Rainfall (100 mm)	1.0020 (0.9884, 1.0156)	0.00026100
		Temperature (°C)	1.0054 (0.9890, 1.0218)	0.00041700
		Humidity (%)	1.0003 (0.9935, 1.0072)	0.00018100
	1996-2000 (3274.24)	Rainfall (100 mm)	1.0002 (0.9889, 1.0114)	0.00022100
		**Temperature (°C)**	**1.0227 (1.0073, 1.0382)**	**0.00038100**
		Humidity (%)	1.0013 (0.9953, 1.0073)	0.00015700
	2001-2005 (3135.13)	Rainfall (100 mm)	1.0086 (0.9920, 1.0252)	0.00033100
		**Temperature (°C)**	**1.0324 (1.0126, 1.0521)**	**0.00051500**
		Humidity (%)	0.9984 (0.9911, 1.0057)	0.00019300
Model III: Socio-economic, meteorological factors and suicide	1986-1990 (3151.85)	**SEIFA**	**0.9985 (0.9972, 0.9997)**	**0.00002250**
		**ATSI (%)**	**0.9912 (0.9843, 0.9980)**	**0.00007530**
		Unemployment (%)	1.0012 (0.9846, 1.0179)	0.00034600
		Rainfall (100 mm)	0.9983 (0.9835, 1.0131)	0.00029300
		Temperature (°C)	0.9974 (0.9752, 1.0196)	0.00057800
		Humidity (%)	0.9948 (0.9862, 1.0033)	0.00022800
	1991-1995 (3179.25)	SEIFA	0.9992 (0.9981, 1.0002)	0.00001780
		ATSI (%)	0.9978 (0.9920, 1.0036)	0.00006770
		Unemployment (%)	1.0071 (0.9921, 1.0221)	0.00028100
		Rainfall (100 mm)	1.0061 (0.9926, 1.0195)	0.00025700
		Temperature (°C)	0.9983 (0.9792, 1.0174)	0.00049300
		Humidity (%)	0.9975 (0.9908, 1.0041)	0.00017200
	1996-2000 (3314.74)	SEIFA	0.9994 (0.9986, 1.0002)	0.00000973
		**ATSI (%)**	**1.0125 (1.0073, 1.0177)**	**0.00006180**
		**Unemployment (%)**	**1.0199 (1.0043, 1.0356)**	**0.00023000**
		Rainfall (100 mm)	1.0095 (0.9942, 1.0248)	0.00030700
		Temperature (°C)	1.0048 (0.9865, 1.0231)	0.00047100
		Humidity (%)	1.0020 (0.9949, 1.0090)	0.00018500
	2001-2005 (3191.78)	SEIFA	0.9995 (0.9987, 1.0004)	0.00001190
		**ATSI (%)**	**1.0101 (1.0055, 1.0148)**	**0.00005560**
		**Unemployment (%)**	**1.0180 (1.0047, 1.0314)**	**0.00020300**
		Rainfall (100 mm)	1.0056 (0.9947, 1.0166)	0.00023100
		Temperature (°C)	0.9991 (0.9834, 1.0147)	0.00039100
		Humidity (%)	0.9990 (0.9925, 1.0054)	0.00016700

SEIFA: Social-Economic Index for Areas. ATSI: Aboriginal and Torres Strait Islanders. This model has fixed effect for each time period**.** All the significant results are in Bold numbers.

## Discussion

This study examined the association of socio-demographic and meteorological factors with suicide at the LGA level in Australia using 20 years data (1986–2005). The spatial patterns of suicide rates varied across different time periods. Socio-environmental variables (e.g., temperature, proportion of Aboriginal and Torres Strait Islanders) appeared to be associated with the occurrence of suicide and socio-demographic variables had stronger associations with suicide than meteorological variables. Identifying significant socio-environmental associations, characterizing these associations over different geographic regions, and determining changes in associations over time, may help researchers to better understand suicide and possibly assist policy makers in designing and implementing more targeted and effective suicide control and prevention strategies. This may also need integration of the knowledge gained from this study and causation of socio-environmental factors to suicide into a multilevel trans-disciplinary analysis in future study.

Given the other variables in the model, the pattern of the associations between the proportion of Aboriginal and Torres Straight Islanders in the population of an LGA and suicide changed dramatically between years before 1990 (negative) and years after 1996 (positive) of the study period. A total of 15 LGAs in QLD, WA and NT had a proportion of ATSI over 50% in the whole study period, compared with less than 0.3% of ATSI among the national population. Between 1986 and 1995, the mean suicide rate (annual average) in these areas was less than 9 per 100,000 (39 cases) and even was lower than the national average. The mean suicide rate in the 15 LGAs, however, increased to over 28 per 100,000 (1996–2005, 158 cases) after 1996 and was over 2 fold of the national average. At the national level, the number and rate of ATSI suicide kept increasing since the early of 1980s while the non ATSI suicide rates in the same period were generally steady [31-33]. There are two major possible reasons for explaining the significant change in the pattern of ATSI suicide on the study period. First, some suicide cases may not be reported, and the non-report rate may be higher among the Indigenous population. Second, Indigenous communities often have social disadvantages, e.g., lower education, worse living conditions, and poor healthcare facilities [34,35]. The introduction of hazard lifestyles (e.g., alcohol use) to ATSI communities caused increased domestic violence and social disruption, and also resulted in psychiatric problems in the communities especially after late 1980s [36]. The intergenerational trauma resulted from colonization and loss of land, language and culture, still remains in the ATSI population [37]. The synthetic effects from above may have triggered and spread suicidal behaviours in the ATSI communities [38,39].

Australia experienced the high unemployment rate nationwide in 1992 and then unemployment rates dropped after that [40]. The results indicate that unemployment rate had more significant associations with suicide between 1996 and 2005 than before. This may due to different ranges of unemployment rate change at the LGAs level over time. The results of positive associations between unemployment and suicide across small areas (1996–2005) are consistent with previous Australian and international studies [9,11].

In this study, temperature has significant association with suicide in Model I, but the association disappeared when socioeconomic factors were controlled in Model III. The results in Model III are generally consistent with a previous study in Taiwan [24] which indicated that socio-economic variables had stronger associations with suicide than climate variables over cities and counties. This indicated that socio-economic factors may mediate the effect of temperature on suicide across different areas. Previous studies reported that increased temperature was associated with higher suicide rates, especially over a time period [3,6]. Increased temperature may reduce the level of serotonin (5-hydroxytryptamine; 5-HT receptors), a concentrating neurotrophic factor which can adjusting central nervous system and affect human mood [41-44]. Anxiety, stress, despair and suicidal behaviors can be triggered by increased temperature. The mean temperature data in each 5-year period used in this study demonstrated the temperature difference across LGAs but did not examine the seasonality of temperature and suicide changes over time. This may mask the potential effect of heat waves on suicide. The impacts of temperature on suicide, however, have become more significant in recent years. Continuing climate change may have increasing impacts on population health, including mental health and suicidal behaviors [1]. Australia experienced increased national annual average temperature from the 1980s till now [45]. This may explain that temperature was more significantly associated with suicide between 1996 and 2005 than 1986–1995 in Model II. As climate changes continue, more frequent and severe natural disasters and extreme weather (e.g., earthquake, drought, hurricane and flood) may damage local environment, communities and properties, and add a financial burden to local population; then anxiety, despair and social disruption may result from disasters and lead to suicidal behaviors [22,46-48]. In this study, most LGAs with high proportion (over 50%) of ATSI population and lower socioeconomic disadvantages are in tropical areas (e.g., top north of NT and QLD), and have higher temperature than other areas. These areas are usually lack of air conditioning systems or electricity support, especially during heat waves [49]. This suggests that high temperature may have more significant impact on deaths, including suicide in ATSI population than non-ATSI population in Australia.

Bayesian CAR model adjusted spatial autocorrelation and uncertainly using covariates and random effects, borrowed strength from adjacent areas and can reduce the risk likelihood which is randomly created [50]. This approach also compensates for spatial variation of residuals resulting from other factors with spatial variation, e.g., population density, home address and psychiatric healthcare facilities, which have also been applied in studies in other diseases [26,51]. Models I and II had lower DIC for each time period than that of Model III, thus Models I and II have better fit. It is difficult to identify the relative magnitude of contribution of meteorological (e.g., temperature) and socio-demographic (e.g., ATSI status) variables to suicide if only using Models I or II, however. Model III, which includes both of meteorological and socio-demographic variables, can address this problem. Thus we keep all three models in the results to elaborate a better understanding of the differences of associations of each variable with suicide in each time periods based on model choice.

This study has three key strengths. Firstly, this is the first study exploring the association of socio-demographic and meteorological factors with suicide across small areas in the whole Australia, using a Bayesian CAR model. Secondly, this study identified the different socio-environmental drivers over various periods and compared the relative contribution of different socio-environmental variables to the patterns of suicide. Finally, the results in this study may have significant public health implications for improving current suicide control and prevention programs to target specific high-risk areas and major socio-environmental drivers.

There are also some limitations in this study. Firstly, detailed personal information of each suicide case, e.g., health status, mental disorders and use of medication before suicide, was not available in the dataset. Therefore, these confounders were not taken into account in this study. Secondly, aggregated socio-environmental variables (5-year average) masked the changes of these variables over time, especially seasonal changes of meteorological variables (e.g., temperature). Thus the association of these variables with suicide over time may be hidden. Finally, underreported cases, especially in the latest years of the study period, may induce bias of underestimated suicide risk in some areas.

Future research needs to include more detailed personal information and examine how associations of socio-environmental factors and suicide varied across different population groups (e.g., sex and age) and suicide methods over time and space. It is important to examine the association between socio-environmental factors and it is interesting to note the dramatic change in the pattern of suicide among Aboriginal and Torres Strait Islanders in recent years. The Bayesian CAR models used in this study may also have potential in examining socio-environmental drivers of other mental health and psychiatric problems.

## Conclusion

This study found that proportion of Aboriginal and Torres Strait Islanders, unemployment and temperature are the main variables statistically associated with of suicide incidence across LGAs among all selected independent variables, especially after 1995. The results indicated that socio-demographic factors outweighed meteorological factors in their associations with suicide across different areas in this study. These data drawn from study are essential for designing suicide control and prevention strategies.

## Competing interests

We declare that we have no competing interests.

## Authors’ contributions

XQ designed the study, implemented all statistical analyses and drafted the manuscript. ST conceptualised the idea and revised the study protocol, especially the research design and data analysis. WH provided advice on statistical analyses and interpretation of the results. KM helped interpreting the results and drafting the manuscript. All authors read and approved the final manuscript.

## Pre-publication history

The pre-publication history for this paper can be accessed here:

http://www.biomedcentral.com/1471-2458/14/681/prepub

## Supplementary Material

Additional file 1: File S1 #Bayesian Spatial CAR model for Suicide in Australia. Click here for file
