# Supplementary material for: Socio-environmental drivers and suicide in Australia: Bayesian spatial analysis
Source: BMC Public Health. 2014 Jul 4;14:681. doi: 10.1186/1471-2458-14-681 (PMC4226967; doi:10.1186/1471-2458-14-681)
Supplement: Additional file 1: File S1 — #Bayesian Spatial CAR model for Suicide in Australia. [file 1471-2458-14-681-S1.doc]

Additional file 1: File S1

#Bayesian Spatial CAR model for Suicide in Australia

Model

{

#Likelihood

for(i in 1:N){

O[i] ~ dpois(mu[i]);

log(mu[i])<- log(E[i]) + log.RR[i];

log.RR[i] <- alpha + beta[1]*(RAIN[i]) + beta[2]*(TEMP[i]) + beta[3]*(SEIFA[i]-935.61)+ beta[4]*(HUM[i])+beta[5]*(IND[i])+beta[6]*(UNE[i])+beta[7]*(AREA[i])+U[i] + S[i];

U[i] ~ dnorm(0,tau.u);

RR[i]<-exp(log.RR[i]);

}

#Priors

S[1:N]~car.normal(adj[],weights[],num[],tau.s);

for (k in 1:SumNumNeigh){

weights[k]<-1

}

tau.u~dgamma(0.01,0.01);

tau.s~dgamma(0.5,0.0005);

alpha~dflat();

beta[1]~dnorm(0,0.00001);

beta[2]~dnorm(0,0.00001);

beta[3]~dnorm(0,0.00001);

beta[4]~dnorm(0,0.00001);

beta[5]~dnorm(0,0.00001);

beta[6]~dnorm(0,0.00001);

beta[7]~dnorm(0,0.00001);

#Functions

sigma.u<-sqrt(1/tau.u);

sigma.s<-sqrt(1/tau.s);

}

#Data

list(N=652, SumNumNeigh=3262)
